# Emotional intelligence with the gender perspective in health organizations managers

**DOI:** 10.1016/j.heliyon.2022.e11488

**Published:** 2022-11-15

**Authors:** Fotis Kitsios, Eumorfia Papageorgiou, Maria Kamariotou, Nikolaos A. Perifanis, Michael A. Talias

**Affiliations:** a Department of Applied Informatics, University of Macedonia, 156 Egnatia Str., 54636, Thessaloniki, Greece; b Faculty of Economics and Management, Open University Cyprus, P.O. Box 12794, Nicosia, 2252, Cyprus

**Keywords:** Emotional intelligence, Health managers, Workforce, Sex

## Abstract

Emotional intelligence (EI) is considered as a necessary qualification for Health Service executives in order to emotionally understand the human resources they manage and how to best handle people, situations and infrastructures. The purpose of this paper is to investigate the EI levels of healthcare executives (senior, middle and junior executives), in the field of health, in relation to sex. An EI Scale Assessment Questionnaire was used, which explores aspects of EI, such as estimating, expressing, regulating and using EI in problem solving. The sample consisted of 161 participants and data analysis was implemented based on criterion *x*^2^. The data analysis showed that health care managers express a high level of emotional perception, and management– evaluation of their own and other emotions. In addition, results in relation to sex revealed that women express higher EI than men and also express higher management-evaluation of self-esteem than men.

## 1 Introduction

On the basis of the research conducted by J. Mayer and P. Salovey [1] , the terms “Emotional Intelligence” (EI) has been defined by H. Gardner [2] and D. Goleman [3] They made the observation that EI consists of not only a characteristic but also an ability that is distinct from common cognitive ability (IQ). EI refers to a person's awareness, control, and expression of their emotions as well as their ability to handle interpersonal relationships in a way that is both wise and compassionate. Even though several definitions of EI have been suggested in the years since Salovey and Mayer were the first to describe it, the premises on which it was founded continue to be central to how the notion is perceived and evaluated in the modern day.

EI or Emotional Quotient (EQ) is considered as the ability of people to combine emotion with intelligence in order to solve problems or make decisions. As a personality trait, or as a human ability, EI is regarded a decisive factor in mental health, enhanced life and professional success [4]. People's capacity to manage their own and other feelings, with the prospect of being useful to society as a whole, provides an opportunity for progress, both individually and collectively [5]. In the workplace, executives, especially staff members, are thought to need more than traditional cognitive abilities and what is required is the skill/ability of empathy/EI in order to reduce negative emotions and focus on positive emotions, leading to the definition of success [6, 7].

Experiencing, recognizing, and expressing feelings was seen as a sign of weakness, a source of uncertainty, and a departure from rationalism and therefore the right decisions. In recent decades, however, things have changed and this view is considered obsolete [8, 9, 10]. It is easy to ignore emotions when working alone in an office, but it is difficult to do so while working as a member of a group where it is natural to create conflicts, disagreements, special ties, sympathies and alliances. Professionals who have a higher EI are able to generate more sales, better manage challenging situations, and maintain their composure even while working under intense time constraints. They are able to effectively lead others and continue to be valued coworkers despite being in a lower position than their counterparts. In recent years, the field of medicine has started to acknowledge the potential advantages of EI, not only for staff but also for patients [11, 12, 13].

In health sector, providing quality services to those most in need, i.e. patients, is considered to be particularly important [[14], [15], [16], [17]]. As stated by researches such as [[18], [19], [20], [21], [22]] on healthcare leadership and healthcare management, EI is noticed as a key element for leading staff executives, but also for middle and lower level executives, in order to be identified and regarded as charismatic and transformational. Overall, there appears to be inadequate research evidence outside the practice of healthcare, in which EI has been correlated with many organizational outcomes to create competitive advantage through leadership efficacy, enhanced retention of top talent, intraprofessional teamwork, effective collaboration and boosted motivation [23, 24, 25, 26]. In the literature though, there seems to be insufficient studies evaluating the ability/competence of EI in health professionals, and especially in executives who hold positions of responsibility (managerial-tactical) in health care organizations [27, 28].

Besides this, no particular attention has been paid to whether the relationship between leadership and emotional characteristics in this field is influenced by sex. Limited surveys have been implemented and investigated the existence of sex differences. However, the analysis of this issue has often led to conflicting results [29]. Although women frequently tend to have a higher score on overall index of EI than men [30, 31, 32], most researches agree on the fact that except for some minor discrepancies in the individual components of EI, there are no standardized differences between men and women.

The COVID-19 pandemic has been particularly dangerous to people's mental and emotional wellbeing. Efforts to slow the spread of the virus have been linked to an uptick in people feeling lonely and isolated and have added many new sources of emotional and financial strain as individuals deal with issues like job loss, reorganizing their careers, and adjusting to other, more unpredictable aspects of their daily lives. The conditions brought on by the COVID-19 pandemic are favorable for the development and maintenance of mental health problems. In light of this, it stands to reason that those with higher levels of EI are better equipped to cope with the emotional problems brought on by the pandemic [33, 34]. There has been a surge in the complexity of healthcare organizations because of the COVID-19 pandemic. Those in positions of authority in the health care industry need to hone their EI abilities so they can handle the increasing demands placed on them [35].

Consequently, the purpose of this article is to examine the level of EI in upper, middle and lower executives employed in health care organizations, with the aim of providing the best possible services to patients. Additionally, it is inspected whether the two sexes working in this field have discrepancies in this characteristic. For this reason, the survey that focused on collecting data on the measurement of EI, referred to in this article, was conducted on executives who hold positions of responsibility (heads of departments, directors, and managers) in five health care providers in a geographical area of Greek territory.

The study focuses on the human factor and is intended to be structured around the following two research questions. Initially, what is the level of empathy or emotional intelligence of the executives who hold positions of responsibility (as mentioned above) in health service organizations in order to be utilized in the most efficient and orderly operation of their area of responsibility? [36, 37, 27, 28, 20, 38]. Furthermore, in which way is sex, as a variable, related to the empathy/EI of administrators in health service units? [[39], [14], [28], [40], [41], [42], [17], [43], [44], [45], [46], [47]].

The contribution of this research to the scientific community is to shed light on the data identified and analyzed in the literature to investigate and record the ability/trait of EI in National Health System management staff, in order to point out its benefits in this everyday demanding workplace. In any case, the present work should lead to the extent appropriate, contribute to the understanding, control and management of the feelings of oneself and others of lower middle, and senior executives working in the health sector, for better labor relations, but also a better and more efficient organization of work. Moreover, the discoveries on sex differentiation can constitute a complementary stone in the unalike aspects of experiencing and understanding of this workplace for women and men. In addition, considering EI and leadership qualities, the aspect of sex would be very exciting because the concept of sex also refers to social roles human occupy and behavioral patterns that are distributed across the spectrum of male and female. Leadership hence may be more gender oriented and influenced aspect.

The structure of the remaining modules is as follows: The first section presents the recording of the research problem, the usage of EI skills/capabilities by chief executives of health service organizations, and the literature review, where findings of previous research are listed, mainly from abroad. The second section focuses on research design, presenting its methodology. Following is the analysis of the survey data, using an EI Scale Assessment Questionnaire (SEIS - AES). The last section lists a discussion of the results in relation to previous studies on the field, pointing the limitations that appeared and some avenues for future research and ends with the most important research conclusions.

## 2 Theoretical background

### 2.1 Emotional intelligence theory

The concept of EI describes people who are characterized by a kind of intelligence related to their emotions and how they are interpreted, which constitutes an important role in people's personal and professional lives [5, 48]. There are currently several models of EI. The two basic models regarding the conceptual approach and measurement of EI are [49] ability model which reflects on the individual's ability to process emotional knowledge and use it to manage the social world and [6] mixed model, which incorporates what was later been modeled separately as EI and traceability. In the early 1990s [48], proposed the existence of a new intelligence considering that it is the ability of one to recognize and manage, not only his feelings, but those of others. Their late model [5] included four main branches (perception, facilitation, understanding and management) with added areas of reasoning possessed by people with high EI. Perception refers to accurate perception/recognition of emotions by the sensory stimuli of ourselves and others (i.e. tone, facial expression, posture or other environmental indicators). Facilitation is the use of emotions and moods to encourage, improve, or enhance work performance. Understanding shows how emotions combine, move forward and change over time and situations. Finally, management refers to managing or regulating the feelings of oneself or other individuals in order to experience a greater positive impact and enhance personal development (both emotional and spiritual).

Goleman [6], due to whom the popularity of the term “EI”, set out a framework for understanding the concept of EI incorporating a set of emotional abilities into each structure of EI. In particular, while the first model presented five main areas [50], distinguish four main categories of EI: self-awareness, self-management, social awareness and relationship management. Self-awareness presents how someone feels. Self-awareness includes emotional awareness, self-esteem, and ultimately self-confidence. Self-management indicates the management of these emotions before letting them “handle” themselves. Self-management consists of emotional self-control, adaptability, goal orientation and positivity. Social awareness refers to how others feel and to be able to handle relationships with them effectively. Social awareness includes subcategories such as empathy, awareness of the whole, and orientation in the service of fellow human beings. Finally, relationship management presents the motivation to complete someone his/her goals, to be creative and to do one's best. Relationship management consists of the following subcategories: leadership, motivation, third party development, conflict management, relationship strengthening, and finally teamwork and collaboration.

When leaders make use of people's emotions, it becomes much simpler for them to consider and inspire the people under their charge through using varying viewpoints that can recognize the correlations among these feelings as well as understand the dynamic emotions and feelings that can change from one circumstance to another. It enables a leader to gain an understanding of what drives people to follow them. Maintaining control of one's emotions enables leaders to better bear the weight of the disappointment caused by actions that result in subpar performance. Leaders who have more effective coping mechanisms are able to deal with the feeling itself rather than the source of the problem, as in the case of denial [27].

In order to measure EI, numerous tests have been developed, which are grouped into two major categories, depending on the method they use to collect information. The Freudenthaler & Neubauer Emotional Intelligence Performance Test (FNEIPT) has been used by Perez, Petrides & Furnham (2005) and the Multifactor Emotional Intelligence Scale (MEIS) has been used by [27] to measure EI through characteristics. Other tests that measure EI are the Assessing Emotions Scale (AES) which has been used by Gourzoulidis [[28], [51], [52], [43], [38]], the Emotional Competency Inventory (ECI) which has been used by [37]; [27], the Emotional Intelligence, the Bar-On Emotional Quotient Inventory (EQ-I) test which has been used by Bar-On [30]; [37]; [14]Tyczkowski et al.[44], the Schutte Emotional Intelligence Scale (SEIS) which has been used by Chan & Hamamura [53] Gourzoulidis et al [[51], [54], [55]]; Wong & Low [56] Emotional Intelligence Test (WLEIS) which has been used by Kafetsios, Nezlek & Vassiliou [40]; Trivellas, Gerogiannis & Svarna, 2013 [57].

The two most used scales of EI in Health sector management [53, 43, 38] appear to be, the SEIS [58] which is fully explained in our study's methodology and the EQ-i 2.0—an assessment tool with a 5-point Likert - type to 133 survey questions based on the five factors based on [59] for EI [37, 14, 44]. Both of the tests belong to the first category which has been used internationally and have sufficient reliability and validity.

On the other hand [60], preferred to use the MSCEIT test, an ability-based assessment, consisted of 141 survey questions and evaluates the 4 components used by [61] EI ability Y based model: perceive, use, understand, and manage emotions in order to associate EI with leadership. Mayer et al. (2000) [61] also mention a third category of tests with an “informant” where a third person is called upon to assess a person's level of EI. This type of test is the Hay-Group Emotional Competence Inventory (ESCI) [62] and the Genos EI Inventory [63] both of which are 360-degree measures, including self-assessment and evaluation by supervisors and subordinates, but not as widely used in scientific research in the field of health services. Other studies such as [14, 17] and [44] have used the Multifactorial Leadership Questionnaire (MLQ) [64], the most well-established research tool for assessing transformational, transactional, and liberating leadership in business.

### 2.2 Emotional intelligence and health sector

Undoubtedly, it is a rhetorical question whether EI could be absent from any capable leader. In order to highlight the correlation between EI and leadership, it is very crucial to also pay attention at the concept of the second component. Specifically, leadership encompasses four distinct competences (capabilities), which are recognized as important components of interpersonal communication-intelligence: (a) the organization of groups, (b) negotiating solutions, (c) interpersonal relationships, and (d) the possibility of social analysis [27, 6, 60]. All these skills together, form the so-called charismatic man. Social intelligence ability is determined by sharpness, emotional perception, leadership and organizational skills, and ease to settle differences that arise in social and work life [27, 65].

Wilderom et al. [22] hypothesize that an emotionally intelligent chief executive is not deemed right to ignore emotion, but must be able to build coherent relationships in the workplace that enhance staff productivity, which in turn it also enhances the objective performance of the health care business, organization or unit. Dulewicz and Higgs [66], the basis of the above, considered that a leader should be distinguished for self-control, intuition, emotional resilience, interpersonal sensitivity, motivation, influence, consciousness and integrity. In a study conducted by [40], findings show that leaders with increased levels of EI interact to contribute to the positive effects of their subordinates, with lower level of EI, on their attitude toward work. This confirms in some way. In several aspects this supports the opinion of [55] that EI, which was originally considered a human trait, can be efficiently reflected in cohesive group characteristics in some instances.

Specifically, a connection between EI and transformational leadership has been explored by several researchers [67, 68, 69]. But there is mixed data. Some authors have discovered valuable connections between EI and leadership in transition [70, 71, 72]. In the healthcare sector, some scholars observed that successful healthcare leadership is necessary because it is deemed vital to a pipeline of burled a pool of healthcare leaders and because effective leadership is key to maximizing healthcare quality, access and efficiency [73]. By pushing themselves and their supporters to accomplish success [68], leaders who use this approach transform organizations and encourage and empower people to support them achieve great results [74]. They are imaginative [68]. Some see transformative leadership as promoting true leadership, imagination and ingenuity while still being able to create trust and partnerships and convey rational care [75].

It is a reasonal view that every organization or business must comply with the proper application of EI at all levels. Abraham [39] and Prufeta [60] believe that EI should be considered as one of the evaluated qualifications of the leadership - staff of health care organizations. Equally important is the continuous training and support of both executives, people at all levels, as well as staff, especially older employees in a hospital. On top of that [37, 14, 27, 40, 60, 17, 44] , investigated the skill/competence of EI in executives - managers and sought to relate it to effective leadership, better management of health staff, so as to lead to more effective and better quality health services, but also to examine the relationship between leadership training and staff.

Wallis and Kennedy [69] discovered that leadership training was important for the continuation of a group, but they also noted that additional research was necessary. There are those who believe that by advancing professional advancement that focuses on enhancing the capabilities of health authorities, the quality of care will improve [76]. Others argue that this type of leadership development is necessary, despite the fact that certain strategies, such as traditional leadership development and communication skills training, are incapable of delivering long-term change in practice [77].

In particular [60, 17], concluded that executives should be trained in the use of empathy/EI and be at the forefront of staff training especially older and more experienced. Feather [27], Spano-Szekely et al. [17] and Tyczkowski et al. [44] highlight the need for of empathy/EI to enhance work performance with the least possible stress, and increased employee satisfaction, while also raising the issue of university staff training to create positive staffing and upgraded patient care. For the same subject, the need to train health professionals and executives, considering the issue as an example of good implementation of transformational leadership in this area [14, 78, 79].

Furthermore [37, 55] acknowledge that emotionally intelligent leaderships in health care units can help their organizations create a supportive work environment through inter-professional teamwork motivation and the creation of innovative methods that promote confidence by creating more efficient work satisfaction conditions, with a direct impact on the quality of provided health services. In fact, the latter, point out that possible applications of training in EI can cover a variety of activities, from education to businesses, in a better understanding of society itself, and many industries should contribute in the examination of the effects that targeted training can have on EI.

To epitomize the meaning of EI on a collective level, it can be considered as a source of valuable knowledge that can help a person to be meaningful and navigated by “reading” his/her social environment, that is appropriate in meeting their daily needs, leading a more effective personal and social life [80]. Due to the aforementioned [81], taking into consideration an earlier study [82, 83], consider sex variable in the case of EI as a way of interpreting the mechanisms of emotional operation. Their research [81] on the connection between sex and EI highlighted an important difference in the measurement of EI in women versus men, noting that a time variable (age) contributes blunts the difference. At the same time [83], have shown that EI in women is considered as an indicator of good mental health, social support, and job support or development.

Remarkable is the research by [28], which carried out in Greece with target population executives of Health Organizations. They collected data from 120 general managers of Greek public health institutions (men and women), who completed a questionnaire for measuring EI with AES scale, in conjunction with the examination of the HRQoL score, through the SF-36. The findings highlighted an important difference in the assessment, regulation and evaluation of emotions by women versus men, with the largest difference being in the assessment/evaluation of emotions, assessing improved judgment and evaluation of their own and other emotions, showing better understanding of non-verbal communication. Kafetsios et al. [40] and Schutte et al. [58] confirm likewise the existence of a sex differences, although it did not carry out in the health field.

Health care services are thought to provide professionals with high levels of EI due to the properties of the sector and the requirement of managing with the reality that the foremost noteworthy issues confronting society are wellbeing related. For that reason, health administrators and managers have to cope with the provision of giving quality services to their clients amid a time of constrained economical budgets and human assets [84]. Having the affectability to perceive human resources issues and act skillfully despite them in a successful way needs managers with high EI. People in administrative positions, despite their sex, have the indispensably significant duty of perceiving the valuable asset they have in their workforce.

## 3 Materials and methods

### 3.1 Research instrument

The data collection methodology was carried out using an anonymous and self-completed questionnaire, using the Emotional Intelligence Scale (SEIS) [58]. The Assessing Emotions Scale AES scale [52], investigates facets of EI and builds on the conceptual basis of [61] model, which focuses on the assessment and expression of feelings, on the regulation of emotions and their use in assessing and resolving problems [28, 58]. Schutte et al. [58] find that the SEIS/AES scale shows good internal reliability, produces consistent results over time and is considered a valid tool for measuring EI. The SEIS-AES scale, in the most complete and most frequently used by researchers version [54], includes 33 self-report statements (questions), focusing on formal EI. Respondents are required to rate the degree to which they agree or disagree with each five-point scale statement/question (1 = Strongly disagree to 5 = Strongly agree). Participants respond on a Likert scale that mathematically evaluates EI. Scores are calculated by the reverse coding items 5, 28 and 33, and then by the sum of all the items. Scores can range from 33 to 165, with higher scores showing greater characteristics of EI [54].

### 3.2 Sample

The sample consists of healthcare managers in five Public Health Service Organizations belonging to the National Health System (NHS) (four General Hospitals and one University General Hospital) in specific geographical region of Greece. Participants were senior, middle, and junior executives who had to meet the following criteria: (a) be an active executive with a position of responsibility (head of department, head, director, administrator) in one of the five organizations providing health services in a specific geographical region of Greece; (b) be of a certain age up to retirement age; (c) have received training commensurate with the position he holds; and (d) there were no gender restrictions. Of the 379 questionnaires, 161 were answered. Informed consent was obtained from all participants for our research.

The descriptive method and the statistical method of inference which was based on criterion *c*^*2*^ (known as the independence test criterion) were used for data analysis. The process was implemented with a statistical test based on the use of the statistical distribution of *c*^*2*^ at significance level [43].

This paper does not involve chemicals; procedures or equipment that have any unusual hazards inherent in their use. Furthermore, it does not include experiments that may raise biosecurity concerns or human subjects or a clinical trial. Finally, the paper does not involve experimentation on animals.

## 4 Results

Table 1 presents the demographic and occupational profiles of the sample of 161 participants. The 128 (79.5%) of the 161 participants in the sample were women while 33 (20.5%) of the 161 participants in the sample were men. Regarding their age distribution, it was observed that 47.8% (*n* = 77) were 46–55 years old and 34.8% (*n* = 56) were 36–45 years old. The lowest participation was seen in individuals in the age groups of 18–25 years (n = 8, 5%), 26–35 years (*n* = 6, 3.7%) and 56 years and over 18 (*n* = 14, 8.7%). The majority of participants were holding a bachelor's degree (*n* = 97, 60.2%) while a significant proportion of the sample were postgraduate or doctoral graduates (*n* = 33, 20.5%). The lowest attendance was observed among secondary school graduates (*n* = 22, 13.7%) and Vocational Training Institutes or technical school graduates (*n* = 9, 5.6%). Finally, regarding to years of experience in administrative positions, it was found that 37.9% (*n* = 61) had administrative experience from one to five years, 23.6% (*n* = 38) had administrative experience from 16 to 25 years, 16.1% (*n* = 26) had experience of management from six to 15 years and 18% (*n* = 29) had experience of management from 26 to 35 years. Also, only seven (4.3%) people had more than 36 years of administrative experience.

**Table 1:** Demographic and occupational sample characteristics.

Demographic and professional sample characteristics		*n*	*%*
**Gender**	Men	33	20.5%
	Women	128	79.5%
**Age**	18–25	8	5.0%
	26–35	6	3.7%
	36–45	56	34.8%
	46–55	77	47.8%
	>56	14	8.7%
**Education**	Secondary Education	22	13.7%
	Technical School	9	5.6%
	BSc Degree	97	60.2%
	Master or PhD Degree	33	20.5%
**Length of service in management posts**	1–5	61	37.9%
	6–15	26	16.1%
	16–25	38	23.6%
	26–35	29	18.0%
	36 +	7	4.3%

### 4.1 Tool reliability analysis

Cronbach's alpha reliability coefficient was used to analyze the reliability of the tool used in the research. The coefficient above 0.7 indicates satisfactory reliability. The results are detailed in Table 2 for both the total scale of EI and the four subscales of EI. The results show that the tool as an aggregate has very high reliability (*α* = 0.901) and all four sub-dimensions have good reliability with alpha values ranging from 0.733 to 0.756. Overall, on the basis of the above, the reliability of the tool is considered to be satisfactory.

**Table 2:** Results of reliability analysis for the overall dimension of EI and its sub-dimensions.

Scale	Number of Questions	Alpha Cronbach
EI	33	0.901
1. Perception of emotions	10	0.744
2. Management – evaluation of emotions of the self	9	0.756
3. Management – evaluation of the feelings of others	8	0.733
4. Using emotions	6	0.735

Below are the results of the sex on the differentiation of EI. A t-test for sex was used for this purpose, shown on Table 3. The analysis revealed that there was a statistically significant difference between men and women in overall EI (*p* = 0.011) and in the dimensions of emotional perception (*p* = 0.032) and self-management of emotional feelings (*p* = 0.032). Whereas men and women expressed equivalent management-evaluation of others emotions (*p* = 0.148) and equivalent use of emotions (*p* = 0.123). More precisely, we observe that women (*A* = 131.48, *SD* = 14.8) express higher EI than men (*A* = 123.91, *SD* = 15.9). Similarly, women (*A* = 38.78, *SD* = 5.1) expressed higher management-evaluation of self-feelings compared to men (*A* = 36.58, *SD* = 5.72).

**Table 3:** Results t-test for differentiation of EI between sexes.

	Gender				*p*
	Men		Women		
	A	SD	A	SD	
1. EI	123.91	15.89	131.48	14.80	0.011∗
2. Perception of emotions	36.58	5.72	38.78	5.10	0.032∗
3. Management – evaluation of emotions of the self	28.7	4.8	31.7	4.5	0.001∗
4. Management – evaluation of the feelings of others	34.33	5.67	35.77	4.91	0.148
5. Using emotions	24.27	3.05	25.24	3.24	0.123

(*A*) Average (*SD*) Standard deviation *p* value < 0.05.

## 5 Discussion

Data analysis showed that senior, middle and junior health executives expressed a high degree of emotional perception, management-evaluation of feelings of self and others, and use emotions. In particular, the average value of the total scale of EI is 129.93 (*SD* = 15.29), considering that the maximum value the scale can get is 165. This value confirms the above analysis.

The responsibility of leaders is to fulfill the needs of staff members by assisting those staff members in developing stronger organizational and leadership skills, while at the same time, the leaders themselves work to become more democratic and humanistic [27]. Effective healthcare organizations place a strong emphasis on developing their leaders' self-awareness and self-management abilities, as well as their psychological and social sensitivity (referred to as EI). Emotional intelligence, or EI, is perhaps the most important hint for healthcare executives on what makes a superior leader. EI refers to the role of the leader in getting people to perform their jobs more successfully [27].

The high value of EI is confirmed by another survey conducted by [53] that highlighted the important role of high emotional skills in personality development and crisis management in the workplace. The results are also confirmed by the study conducted by [37], who argued that skills of EI are considered to be an indicator of inspirational leadership in health care units and may lead to a qualitative upgrade of the health services provided. Moreover [27, 20, 38], point out the scientific basis of EI in the development of staff, middle and junior executives, in the recognition of feelings of self and others, but also in the ability to read and understand nonverbal forms of communication.

Leaders in health management who have developed EI typically have a strong awareness of how important people are to their own personal and professional health, a genuine appreciation for the contribution that people make, and a strong desire to promote a healthy culture that facilitates the exchange of information, the making of decisions, and the appropriate management of emotions. They frequently encourage not only themselves but also the people who report to them to think about awareness and learning in order to improve their leadership abilities and provide an opportunity for them to discover both their strengths and their weaknesses. In this way, they are able to support knowledge and innovation while also developing mutually beneficial working relationships. As mentioned by [67], these relationships are necessary to facilitate the use of knowledge that leads to standard health practice. Emotionally intelligent leaders are distinguished from other leaders not only by their ability to inspire and motivate others but also by their enthusiasm for health care services, their drive for excellence, and their commitment to excelling in all that they do while utilizing all of their skills to motivate passion and excellence. Emotionally intelligent leaders are able to manage conflict and communicate empathy to their staff or families through their use of emotional skills. They have the potential to have a beneficial effect on highly stressful clinical environments, as is made abundantly clear by [85].

The analysis revealed that there was a statistically significant difference between men and women in overall EI (*p* = 0.011). Such findings are reported in many studies conducted by [86, 87, 81, 28, 83, 40, 42, 4, 43]. The research implemented by [58] also found that women tend to have higher level of experience in emotional expression than men.

Also, noteworthy is the difference in the dimensions of women's perception of emotions (*p* = 0.032), but also in the management - evaluation of feelings of self (*p* = 0.001). On the other hand, men and women express equivalent management-evaluation of others' emotions (*p* = 0.148) and equivalent use of emotions (*p* = 0.123). The above findings confirm research by [51, 88, 4], which may offer a reason for the higher degree of women's perception of other people's feelings. Furthermore, conclusions highlighted by [89] noted that the statistical disparity found in the above mentioned cases of EI in favor of women may be due to the way in which society grows young people, based on racial stereotypes and racial prejudices, in order to consider their emotions and interpersonal relationships, becoming more emotional and expressive. After all, it is observed that the reported sex difference in emotional sensitivity (EI), if considered as a valid item, further emphasizes the importance of emotional aspects in the workplace [40].

Explanatory to the above results, previous research by [31, 90] has shown that women excel men in recognizing and understanding emotions, empathy, social adjustment, and interpersonal communication. Women show a stronger perception of emotion than men [82, 90, 56] and self-knowledge [91], while also showing significantly better interpersonal skills, as they are more capable of coding and decoding verbal communication data than men, but also communication skills with an emphasis on service [91].

Women tend to pay more attention to visual stimuli/habits, especially facial expressions, are more expressive than men, and are more able to express emotions on their faces, smiling more often and generally using their facial expressions to show their emotions. In general, men tend to be more open to women than men. Women often appear to be more extroverted, and are considered stronger companions than males. Still, women can provide better emotional and social support than men. Men, on the other hand, are better at handling feelings such as fatigue better at adjusting to new circumstances, and are more constructive and hopeful than women [30, 61]. They also have greater self-confidence and optimism [30], adapt more easily to change, and better handle stress [30, 50]. Women are more characterized by social responsibility, while men are more characterized by adaptability to new conditions [91]. In any case, it should be borne in mind that sex, as a factor that explains the behaviour of the individuals, always operates in complex interactions with other factors such as demographic and socio-cultural [92].

In addition, studies implemented by [14, 60, 55] indicate that age, level of education/training and years of administrative service do not have a positive or negative effect on the levels of empathy/EI, emphasizing the need to train not only executives but also of all employees in the skills of empathy/EI, with the ultimate goal of providing better quality services in the field of health organizations.

The predominance of women is characteristic in the overall EI, but also especially, in the interpretation of feelings and in the management - evaluation of the emotions of the self as opposed to men. This fact should not exacerbate the disparities between the two sexes in this specific professional sector as both use emotions and evaluate the emotions of others at the same level.

## 6 Conclusions

### 6.1 Limitations

As the paper was based on self-assessment and not a combination of evaluating an evaluator or collecting 360-degree data process, the findings are more questionable as they depend on the level of self-awareness of the respondents and may be influenced by misconceptions about themselves and social recognition factors. Another important limitation is the fact that this sample only includes individuals who were willing to respond to a random survey with no compensation and who were in Greece. It is possible that EI is simply higher in this group of individuals who were willing to take the survey than it is in the management population at large. Thus, the results cannot be generalized.

Another limitation refers to the extremely unbalanced experimental group in relation to the main factor: the difference between men and women. However, it can be assumed that the profession of the experimental group is mainly attended by women. A balance is extremely difficult to achieve here and therefore understandable. For empathy and EI, there are a number of studies conducted in Greece, but they are not compatible with the research questions of the paper. In addition, as the study is before COVID 19, it is fruitful to mention that these results may differ under the pandemic regime and it would be particularly interesting to repeat the measurements today in order to compare the results and effects between the two time periods.

### 6.2 Future research

This research can provide the requisite documentation for future studies, especially in Greece, exploring both the variables of the present study but also other additional variables, such as age, education level, leadership styles, the number of patients in different organizations, the relationship of leading executives or employees in contact with patients and attendants in the emergency departments or other departments of health organizations, in order to examine all the aspects of EI in the health sector.

In addition, as the research was chronologically and geographically demarcated by a single survey at a particular period and limits the generalizability of the results, we believe that the survey could be replicated at another point in the same location, using the same target population, with the same research tool, to a comparative study of the data of the two surveys over the course of time, with the prospect of drawing noteworthy conclusions. Likewise, a different method of analyzing the data could be used, or parallel research (using the same or different variables) could be carried out in collaboration with other countries' hospitals to produce useful and comparable results. Moreover, it is suggested to use regression analysis to investigate the impact of sex on the EI of managers in healthcare organizations based on controlling other demographic variables such as age or education level. Finally, future researchers could consider comparing executive groups across senior, middle, and junior executive groups.

Due to the widespread spread of COVID-19, healthcare systems have become more intricate than ever. Leaders in the health care industry need to hone their EI abilities in order to handle the increasing demands placed on them. Therefore, healthcare managers are urged to exhibit EI when interacting with their teams. This includes using their personal and interpersonal competencies to promote self-awareness of their status, attention to squad emotions, self-perception of their interaction style, and understanding of the team, collaboration and teamwork, and assistance towards a new vision. A new approach to incorporating EI into healthcare operations should be developed in light of the aforementioned concepts. Taking into account the difficult circumstances that healthcare leaders around the world must deal with, EI must be implemented and training is crucial. In the future, researchers can bolster the clinical implications of EI and EI training by investigating whether or not those who take part in EI training programs are able to acquire the emotional skills necessary to ensure the proper, fully comprehend, and enforce their negative emotions and whether or not these abilities are critical for mitigating mental health problems associated with the pandemic.

## Declarations

### Author contribution statement

Fotis Kitsios: Conceived and designed the experiments; Performed the experiments; Analyzed and interpreted the data; Contributed reagents, materials, analysis tools or data; Wrote the paper.

Eumorfia Papageorgiou: Performed the experiments; Contributed reagents, materials, analysis tools or data.

Maria Kamariotou: Contributed reagents, materials, analysis tools or data; Wrote the paper.

Nikolaos A. Perifanis: Contributed reagents, materials, analysis tools or data.

Michael A. Talias: Conceived and designed the experiments; Analyzed and interpreted the data.

### Funding statement

This research did not receive any specific grant from funding agencies in the public, commercial, or not-for-profit sectors.

### Data availability statement

The authors do not have permission to share data.

### Declaration of interest's statement

The authors declare no conflict of interest.

### Additional information

No additional information is available for this paper.

## Appendix A Questionnaire

I know when to speak about my personal problems to others.

When I am faced with obstacles, I remember times I faced similar obstacles and overcame them.

I expect that I will do well on most things I try.

Other people find it easy to confide in me.

I find it hard to understand the non-verbal messages of other people.

Some of the major events of my life have led me to re-evaluate what is important and not important.

When my mood changes, I see new possibilities.

Emotions are one of the things that make my life worth living.

I am aware of my emotions as I experience them.

I expect good things to happen.

I like to share my emotions with others.

When I experience a positive emotion, I know how to make it last.

I arrange events others enjoy.

I seek out activities that make me happy

I am aware of the non-verbal messages I send to others

I present myself in a way that makes a good impression on others.

When I am in a positive mood, solving problems is easy for me.

By looking at their facial expressions, I recognize the emotions people are experiencing.

I know why my emotions change.

When I am in a positive mood, I am able to come up with new ideas.

I have control over my emotions.

I easily recognize my emotions as I experience them.

I motivate myself by imagining a good outcome to tasks I take on

I compliment others when they have done something well.

I am aware of the non-verbal messages other people send.

When another person tells me about an important event in his or her life, I almost feel as though I experienced this event myself.

When I feel a change in emotions, I tend to come up with new ideas.

When I am faced with a challenge, I give up because I believe I will fail.

I know what other people are feeling just by looking at them.

I help other people feel better when they are down.

I use good moods to help myself keep trying in the face of obstacles.

I can tell how people are feeling by listening to the tone of their voice.

It is difficult for me to understand why people feel the way they do.
